# Supplementary material for: Specific post-translational modifications of soluble tau protein distinguishes Alzheimer’s disease and primary tauopathies
Source: Nat Commun. 2023 Jun 22;14:3706. doi: 10.1038/s41467-023-39328-1 (PMC10287718; doi:10.1038/s41467-023-39328-1)
Supplement: Supplementary file 1 — Supplementary Information [file 41467_2023_39328_MOESM1_ESM.pdf]

Specific post-translational modifications of soluble tau protein distinguish between Alzheimer’s disease and primary tauopathies.

SUPPLEMENTARY INFORMATION

**Supplementary Table 1 | Insoluble tau isoforms absolute quantities (moles)**			
Disease	Subject #	4R	3R
AD	1	1,1599E-13	1,204E-13
AD	13	2,8871E-13	4,1685E-13
AD	14	1,1606E-13	1,4278E-13
AD	15	1,7004E-13	1,2841E-13
AD	2	3,622E-14	4,4873E-14
AD	3	7,612E-14	8,4628E-14
AD	5	9,7738E-14	1,1541E-13
CBD	16	1,5949E-13	3,3947E-15
CBD	17	1,6345E-13	2,9716E-15
CBD	18	1,1556E-13	1,589E-15
CBD	19	2,2114E-13	2,5802E-15
CBD	44	3,3189E-13	1,6315E-15
PICK	20	2,2693E-15	3,8911E-14
PICK	21	3,1725E-15	1,9845E-14
PICK	22	3,5512E-15	1,5622E-14
PICK	23	1,902E-15	7,662E-14
PICK	24	2,9509E-15	6,139E-14
FTLD	25	2,5813E-15	3,516E-14
FTLD	26	2,3655E-15	1,3671E-14
FTLD	27	1,1399E-13	2,5695E-15
FTLD	29	2,92E-15	2,7071E-14
FTLD	30	1,2959E-14	4,4113E-14
FTLD	31	8,5068E-14	3,7104E-15
FTLD	32	4,6417E-15	1,0731E-13
FTLD	35	2,0433E-13	2,5937E-15
FTLD	36	2,6094E-15	3,7565E-14
FTLD	37	8,0715E-15	2,7693E-15
CTL	39	7,86E-15	4,7085E-15
CTL	40	5,073E-15	4,5318E-15
CTL	41	5,3977E-15	4,4712E-15
CTL	42	3,1525E-15	2,2349E-15
CTL	43	5,3376E-15	2,9088E-15

Supplementary Table 1 | Targeted MS proteomic (SureQuant absolute quantification) Sarkosyl-insoluble tau 4R and 3R isoforms quantification results.

Supplementary Table 2 | Soluble tau isoforms absolute quantities (moles)			
Disease	Subjects #	4R	3R
CTL	39	4,75284E-13	3,6985E-13
CTL	40	9,56426E-14	8,93661E-14
CTL	41	1,23719E-14	1,59865E-14
CTL	42	3,7237E-13	3,30028E-13
CTL	43	6,16071E-13	4,61233E-13
AD	1	3,07834E-13	1,7686E-13
AD	10	4,70941E-13	3,0684E-13
AD	11	3,47575E-13	2,16682E-13
AD	12	4,58822E-13	2,38862E-13
AD	13	6,57277E-13	3,89077E-13
AD	14	3,59392E-13	1,83197E-13
AD	15	4,35037E-13	2,96399E-13
AD	2	4,02214E-13	2,24747E-13
AD	3	6,16071E-15	4,03264E-15
AD	4	5,02401E-13	3,46015E-13
AD	5	2,6885E-13	1,45103E-13
AD	6	2,38399E-13	2,84733E-13
AD	7	4,93362E-13	3,33197E-13
AD	8	5,25782E-13	3,43566E-13
AD	9	5,54161E-13	3,80004E-13
CBD	16	5,02704E-13	2,43038E-13
CBD	17	6,67074E-13	3,33413E-13
CBD	18	4,64629E-13	1,36606E-13
CBD	19	5,03916E-13	1,7938E-13
CBD	44	3,27427E-13	1,66346E-13
FTLD	25	4,74223E-13	2,17042E-13
FTLD	26	2,68648E-13	1,65122E-13
FTLD	27	4,28776E-13	1,81037E-13
FTLD	29	2,41429E-13	2,21075E-13
FTLD	30	2,40217E-13	1,60513E-13
FTLD	31	4,58266E-13	5,15745E-13
FTLD	32	4,42915E-13	2,56792E-13
FTLD	35	8,44573E-13	4,38981E-13
FTLD	36	1,85276E-13	1,34733E-13
FTLD	37	3,79843E-13	4,42366E-13
PICK	20	3,83681E-13	2,49303E-13
PICK	21	4,64831E-13	4,01391E-13
PICK	22	3,43233E-13	1,88598E-13
PICK	23	2,47136E-13	2,60609E-13
PICK	24	2,53246E-13	1,79308E-13

Supplementary Table 2 | Targeted MS proteomic (SureQuant absolute quantification) Sarkosyl-soluble tau 4R and 3R isoforms quantification results.

Supplementary Table 3 | Stable isotope labelled synthetic peptides				
**Tau isoform**	AQUA-peptides amino acid sequences	HPLC Purity	MW (Da)	m/z
3R	VQIVYKPVDLSK (+8)	98 %	1388.67	466.281
4R	HVPGGGSVQIVYKPVDLSK (+8)	98 %	1980.29	663.373
0N	AEEAGIGDTPSLEDEAAGHVTQAR (+10)	98 %	2424.52	812.050
1N	STPTAEAEEAGIGDTPSLEDEAAGHVTQAR (+10)	98 %	3011.12	1007.470
2N	STPTAEDVTAPLVDEGAPGK (+8)	98 %	1955.11	981.990

Supplementary Table 3 | Stable isotope labelled synthetic peptides. AQUA-grade isotopically labelled peptides selected as specific to tau protein isoforms were synthesized by Synpeptide Co Ltd (Shanghai, China 201204) and used as internal standards for absolute quantification. The peptide sequences were made with heavy c-terminal amino-acid carrying 13C and 15N stable isotopes. Molecular weight (MW, Daltons (Da)) and mass/charge ratios (m/z) of each peptide are shown. HPLC, high performance liquid chromatography.

Supplementary Table 4 |Table of all identified Tau PTMs

**Kruskal Wallis FDR adjusted P-Value**		**Wilcoxon** group pairwise comparison (**P* < 0.05; ***P* < 0.005 ; ****P* < 0.0005)														
		**AD** vs CTL	**CBD** vs CTL	**FTLD 4R** vs CTL	**FTLD 3R** vs CTL	**PiD** vs CTL	**AD** vs CBD	**AD** vs FTLD 4R	**AD** vs FTLD 3R	**AD** vs PiD	**CBD** vs FTLD 4R	**CBD** vs FTLD 3R	**CBD** vs PiD	**FTLD 3R** vs FTLD 4R	**FTLD 4R** vs PiD	**FTLD 3R** vs PiD
Sarkosyl-**Insoluble** tau (aggregates)																
**Ub-K311**	**2E-04**	++					++	++	++	++						
**P-S262**	**1E-03**	++					++	+	++	++		+				
Ac-K281	2E-03						-	-				-	-			
P-T217	4E-03	++	++					+	++	++		++	+			
P-S396	4E-03	+					+		+	+						
P-S324	4E-03	++	++	+	+			+	++	++		++	+			
P-T231 + P-S238	8E-03	++	++	+				++	++	++	+	++	+			
**Ub-K369**	**9E-03**		+				- -				+	++	+			
Ub-K343	9E-03	+	++						+			++	+			
Ac-K353	2E-02	+							+	+						
**Ac-K311**	**2E-02**	+			+		+	+				-				
P-S396 + P-S400	3E-02							+	+	+		+	+			
Ac-K369	4E-02	+								+						
P-T231	4E-02	++	++	+	+	+		+	+	+						
**Met-K331**	**4E-02**				+							-				
P-T212 + P-T217	5E-02															
P-T181	5E-02															
P-T319	5E-02															
Met-K369	4E-01															
Ub-K317	6E-01															
Sarkosyl-**Soluble** tau																
Ub-K254	4E-04	++	++					++	+++	++	+	++	++			
**Ub-K311**	**4E-04**	++					++	++	++	++						
Ub-K259	4E-04	++	+			-		+	+++	++		++	++		+	
P-S262	4E-04		++	+	++		- - -	-	- - -							
**P-S262 + Ub-K267**	**4E-04**	++					++	+	++	++						
**Ub-K317**	**4E-04**	++					+	++	++	++						
Ub-K267	4E-04	++	++	+		+	+		++	++		++	++			
**Ub-K369**	**1E-03**		++				- - -				+	++	++			
**Ub-K343**	**1E-02**		+				- -		+		+	++	++	-		-
P-T231 + P-S237 + P-S238	2E-02	++	++	+	++	+		+			+					
P-S396 + P-S400 + P-S404	2E-02	++	++	+	++						+		+			
Ub-K311 + Ub-K317	2E-02								+							
**Ac-K311**	**3E-02**	+			+	+	+					-	-	+	-	
P-S184 + P-S185	3E-02	++		+	+	+	+									
P-S113	4E-02	+		+	++				-							
P-T212 + P-T217	4E-02	++	++		++											
Met-K180	4E-02	++	+	+	+	+										
Ub-K274	4E-02								++	+		+				
P-S396 + P-S400	4E-02	+	+	+	++	+										
P-T39 + P-T50	5E-02															
P-S185 + P-S191	5E-02															
Met-K331	5E-02															
P-T231 + P-S238	5E-02															
Ub-K375	6E-02															
P-S396	6E-02															
Met-K150	6E-02															
Met-K267	6E-02															
P-S46	7E-02															
P-S324	7E-02															
P-T175	1E-01															
P-S404	2E-01															
Met-K174	2E-01															
P-T175 + P-T181	2E-01															
Met-K385	2E-01															
Met-K317	3E-01															
P-S64	3E-01															
Met-K369	3E-01															
P-S422 + P-S433 + P-S435 + Ub-K438	3E-01															
P-T231	4E-01															
P-T181	4E-01															
Ac-K24	4E-01															
Met-K259	4E-01															
P-S202	9E-01															

Supplementary Table 4 | Sarkosyl soluble and insoluble Tau PTMs (complete table). This table is similar to Table 2 but also includes identified PTMs that were not different between controls and diseased conditions (FDR adjusted Kruskal-Wallis's P ≥ 0.05). Right panel display the result of the post-hoc Wilcoxon's pairwise group comparisons on PTMs (+/- P < 0.05; ++/- - P < 0.005; +++/- - - P <0.0005). All statistical tests were two-tailed. Plus sign and Minus sign represent either an increase or decrease, respectively, for the conditions highlighted in bold characters in the panel heading. Bold font, PTMs specific for AD (insoluble tau Ub-K311 and P-S262; soluble tau Ub-K311, Ub-K317 and P-S262+Ub-K267), for 4R-tauopathies (insoluble tau Ub-K369; soluble tau Ub-K343 and Ub-K369) and for 3R-tauopathies (insoluble tau Ac-K311 and Me-K331; soluble tau Ac-K311). PTMs are numbered according the full-length (2N4R) tau protein. AD, Alzheimer's disease. (n=15); CBD, corticobasal degeneration (n=5); PiD, Pick's disease (n=5); FTLD, frontotemporal lobe degeneration (n=10, including FTLD-4R, n=4 and FTLD-3R, n=6); control subjects, CTL (n=5). Source data are provided as a Source Data file (see sheet 2 for the abundances values and sheet 4 for the Wilcoxon test's exact *P*-values).

Supplementary Table 5 | Critical resources		
REAGENT/RESOURCE	ORIGIN	IDENTIFIER/DETAIL
**Antibodies/crosslinker**		
HJ8.7 anti-tau *mouse* monoclonal antibody (Epitope: ^{118}**AAGHV**^{122}, 2N4R full-length tau)	David Holtzman (Washington University School of Medicine; Missouri; USA)	Cat # HJ8.7, RRID: AB_2721234
Tau (D1M9X) XP® *rabbit* monoclonal antibody	Cell Signaling Technologies	Cat # 46687
Anti-vinculin (clone VIN-11-5)	Cytoskeleton, Inc.	Cat # AVN01
Dimethyl pimelimidate dihydrochloride (DMP)	Sigma-Aldrich	Cat # D8388
**Biological samples**		
Human brain tissue	Netherlands Brain Bank (NBB)	See Table 1
**Chemicals, Recombinant protein, synthetic peptides**		
AQUA-grade isotopically labelled peptides	Synpeptide Co Ltd Shanghai, China 201204	See Supplementary Table 3
Recombinant Tau_{441}	Isabelle Landrieu (*Institut Pasteur de Lille*, France)	N/A
Formic acid (FA)	Merck	Cat # 100264
Trifluoroacetic acid (TFA)	BIOSOLVE	Cat # BIO-20230601
Water	VWR	Cat # 23595.328
Acetonitrile (ACN)	VWR	Cat # 83639.320
Roche cOmplete, EDTA-free	Roche	Cat # 05892953001
Triethylammonium bicarbonate (TEAB)	Thermo Scientific™	Cat # 90114
Protein G Sepharose® 4 Fast Flow	GE Healthcare	Cat # 17-0618-01
Phosphatase, Alkaline–Agarose from calf intestine	Sigma-Aldrich	Cat # P0762
Sequencing grade modified Trypsin	Promega	Cat # V5111
**Commercial Assays**		
Pierce™ BCA Protein Assay Kit	Thermo Scientific™	Cat # 23225
Pierce™ Quantitative Colorimetric Peptide Assay	Thermo Scientific™	Cat # 23275
**Lab Equipment**		
Protein LoBind® Tubes	Eppendorf	Cat # EP0030108116 / # EP0030108132
Orbitrap Fusion™ Lumos™ Tribrid™ Mass Spectrometer	Thermo Scientific™	Cat # IQLAAEGAAPFADBMBHQ
Open-Top Thickwall Polycarbonate Tube	BECKMAN COULTER	Cat # 355645
**Software and algorithms**		
R version (version 4.2.2)	R project	https://www.r-project.org/
Skyline (version 21.1)	MacCoss Lab Software, University of Washington, Seattle, WA	MacLean et al., 2010
Proteome Discoverer (version 2.5)	Thermo Fisher Scientific, USA	N/A
**Thermo Scientific™ SureQuant Tune (**Version 3.5)	Thermo Fisher Scientific, USA	N/A

Supplementary Table 5 | Critical resources used to conduct the experimental procedures.

Supplementary Table 6 | SureQuant targeted mass trigger	
**Tau isoform 3R**	**VQIVYKPVDLSK**
Precursor [M+1]	466.615
Precursor [M+2]	466.949
y6	666.391
b2	228.134
y8	957.549
y2	242.159
**Tau isoform 4R**	**HVPGGGSVQIVYKPVDLSK**
Precursor [M+1]	663.707
Precursor [M+2]	664.041
Precursor [M+3]	664.376
b2	237.134
y8	957.549
y17	876.492
**Tau isoform 0N**	**AEEAGIGDTPSLEDEAAGHVTQAR**
Precursor [M+1]	812.384
Precursor [M+2]	812.718
Precursor [M+3]	813.052
b2	201.086
y15	795.890
y7	778.419
**Tau isoform 1N**	**STPTAEAEEAGIGDTPSLEDEAAGHVTQAR**
Precursor [M+1]	1007.804
Precursor [M+2]	1008.138
Precursor [M+3]	1008.472
y11	1164.563
y15	795.890
y7	778.419
**Tau isoform 2N**	**STPTAEDVTAPLVDEGAPGK**
Precursor [M+1]	982.492
Precursor [M+2]	982.993
y3	309.201
y12	1162.619
y10	990.534
y7	681.329

Supplementary Table 6 | SureQuant targeted mass trigger. Left: List of experimental transitions obtained for heavy AQUA peptides and used to trigger high sensitivity MS quantitative MS2 scan for endogenous specific Tau-isoforms peptides. Right: experimental mass/charge ratios (m/z) of each transition.

NETHERLANDS BRAIN BANK

Meibergdreef 47
1105 BA Amsterdam
The Netherlands

T +31 20 566 54 99
F +31 20 691 84 66
www.brainbank.nl

Ethical and legal declaration of the Netherlands Brain Bank

This document summarizes the ethical principles abided by the Netherlands Brain Bank (NBB) and describes legal embedding of the procedures of the NBB. You can cite or use this document when submitting project proposals for review by a designated ethics committee, research council or in proposals for Framework projects financed by the European Commission.

Information and consent
All Material and Data collected by the NBB are obtained on the basis of written informed consent. Where it concerns persons who, for reasons of their health, are unable to give informed consent (incompetent persons), informed authorization is obtained from the legal representative as defined in the Netherlands Civil Code (Burgerlijk Wetboek).

Informed consent/ authorization explicitly permit:

a. To perform an autopsy during which the brain and optionally spinal cord will be removed, in accordance with the Burial and Cremation Act (Wet op de Lijkbezorging);
b. To store and distribute the tissue to scientific research projects reviewed by the scientific committee of the NBB;
c. To use anonymized donor data for the purposes of scientific research.

Registration as a donor is voluntary, without any payment or undue incentives.

Post-mortem procedures and subsidiarity of brain donation
At the time of donor's death the NBB is notified by the next of kin or a designated person. Although rapid autopsy protocol is practiced, the family is always consulted as to the details of transportation and autopsy.

Autopsies for brain donation are only performed when the person does not qualify as an organ donor in terms of Organ Donation Act (Wet op de Orgaandonatie). Where it concerns unnatural causes of death[1], brain autopsy is only performed when the body has been released by the public prosecutor (Officier van Justitie).

All autopsies are performed at the designated premises of the VU Medical Center in Amsterdam. Diagnostic examination and dissection of the central nervous system organs are performed by pathologists registered in accordance with Individual Health Care Professions Act (Wet BIG). The body of the deceased is restored by trained professionals (autopsy assistants).

---

1 This includes the cases of Euthanasia or physician assisted suicide, which can be legally performed in the Netherlands subject to fulfilment of conditions laid down in Termination of Life on Request and Assisted Suicide (Review Procedures) Act (Wet toetsing levensbeëindiging op verzoek en hulp bij zelfdoding).

NETHERLANDS BRAIN BANK

Meibergdreef 47
1105 BA Amsterdam
The Netherlands

T +31 20 566 54 99
F +31 20 691 84 66
www.brainbank.nl

Privacy
Any data collected about the donors and next of kin is processed in accordance with the General Data Protection Regulation (Algemene Verordening Gegevensbescherming, since May 25 2018, before that the Wet Bescherming Persoonsgegevens) and the principle of medical confidentiality (World Medical Association, The International Code of Medical Ethics, 1949). The data protection authority has been notified of the processing of personal data by the NBB. Employees of the NBB who are granted access to the identifiable information have been informed about the obligations to keep such data confidential and have signed a nondisclosure agreement.

The material distributed to researchers is accompanied by anonymized donor information. **The recipient of the tissue is never granted access to identifiable information of the donor.** The researcher is not allowed to carry out any procedures by which the identity of the donor could be derived.

Safety
All tissue recipients are informed that the material has not been tested for infectious agents and should always be handled as potentially hazardous. Researchers are informed of the safety methods in handling the material.

Fair and Regulated Distribution
Material and Data are only distributed on the basis of approved application, disclosing the relevant research project details, including the amount and nature of the requested material and proposed use of the material. All applications for NBB tissue for research projects are evaluated by a scientific committee, on feasibility and scientific quality.

Tissue is transferred under the conditions of the Material Transfer Agreement, which restricts the use of the material to approved research projects only and forbids utilizing material for commercial purposes.

All recipients are responsible to return unused samples to the NBB and dispose of tissue rests according to local safety regulations.

Miscellaneous
Procedures, information - and consent forms of the NBB have been approved by the Medical Ethics Committee of the VU Medical Centre at April 30, 2009.

Dr. I. Huitinga, Director of the Netherlands Brain Bank
Amsterdam, September 21, 2009

Immuno-precipitation

Not recommeded

ELISA

1:20,000

Not determined

4. ELISA (Not determined)

Reagents required
1. Protein binding plate (e.g. Costar cat. # 9018).
2. Multi-channel pipettor
3. Peroxidase developing reagent (e.g. Sigma cat. # P 9187).
4. Secondary antibody conjugated to HRP (Cytoskeleton cat. # MG04)
5. PBS (no azide)
6. Non-fat dried milk
7. 50 mM carbonate buffer at pH 9.0

Method:
1. Use detergent rich extraction buffer to solubilize the talin.
2. Dialyse or dilute out any detergents from the antigen using mini-dialyzers and PBS buffer, to a concentration of less than 0.05% w/v detergents.
3. Pipette 50ul of antigen directly into the bottom of the well and incubate with a lid 2h at 24C or 4C overnight.

After 2h or Next day,
1. Aspirate antigen
2. Block well surfaces with 300 or 330ul of 5% non-fat dried milk in PBS, volume needs to be up to the top of the well so adjust accordingly. Incubate at 24C for 2h.
3. Aspirate block agent
4. Wash three times with PBS 300ul each for 5min each, shake out after each wash and tap upside down on paper towels.
5. Incubate in 100ul of 1/5,000 AVN01 in PBS for 1h at 24C
6. Aspirate primary antibody, and wash three times with PBS 300ul each for 5min each, shake out after each wash and tap upside down on paper towels.
7. Incubate in 200ul of 1/25,000 anti-mouse-HRP (cat. # MG04) plus 1% non-fat dry milk in PBS, for 2h at 24C.
8. Wash four times in TBST 300ul each for 5min each, shake out after each wash and tap upside down on paper towels.
9. Incubate in 1x developing agent for 20-60 min at 37C, record the incubation time.
10. Read absorbance at 405nm and tabulate the results.

The Protein Experts Cytoskeleton

Anti-Vinculin
(Mouse monoclonal Vin-11-5)

Cat.# AVN01
Lot# 025 **Store at -20°C**

Material

AVN01 is provided as an affinity purified mouse monoclonal antibody. The antigen was native vinculin. The preparation is useful for western blot analysis where there is one band in most mammalian, avian and amphibian cell extracts with a molecular weight of 140Kdal. The positive control is liver extract (Cat# EXT06). The antibody is also useful for in situ staining where it will detect specific structures which contain vinculin e.g. focal adhesions.

Storage and Handling

AVN01 should be stored at 4C before reconstitution. The material appears as a white solid that should be resuspended in 200ul of PBS plus 30% glycerol and then stored at -4C for upto two weeks. Longer storage can be achieved by freezing aliquots in liquid nitrogen and storing at -70C. Resuspend the positive control in 0.5ml of Milli-Q water for a final concentration of 2ug/ul total protein and store in aliquots at -20C.

Western Analysis

1:1,000

1 2 3 4 5

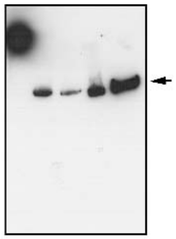

Western blot performed with 1:1,000 of AVN01. Chemiluminescence detection with the Chemiluminesence Detection Kit from KPL Inc. Arrow indicates vinculin at 140Kdal. Lane 1- MWM markers, Lane 2- brain extract, Lane 3- thymus extract, Lane 4- liver extract, Lane 5- HT1080 cell line extract.

Immuno-cytochemistry

1:200

Not available at the time of printing.

Legend: In situs are performed with 1:200 of AVN01. Primary antibody was detected with goat anti-mouse rhodamine conjugate at 1:500.

Product Uses

* Western analysis.
* Immuno-cytochemistry.
* Immuno-precipitation
* ELISA.

Methods

1. Western analysis

Materials required:

1. Run samples on a 6% or 4 to 12% gradient PAGE-SDS gel to 5mm from the end of the gel.
2. Blot proteins onto a PVDF membrane (Millipore Inc.) using 0.25% SDS, 20% methanol in a Tris-Glycine buffer and a 2h blotting run at 300mA (Biorad Mini-blot apparatus).
3. Block for 45 min in 5% non-fat dry milk in 20mM Tris-HCl pH8.0, 50mM NaCl, 0.01% Tween20(TBST).
4. Probe with 1/1,000 of AVN01 in TBST + 1% non-fat dry milk for 1h at RT. 1/500 can be used for low abundance samples.
5. Wash 3 X with TBST for 10min each.
6. Probe with 1/50,000 anti-mouse-HRP (cat. # MG04), plus 1% non-fat dry milk in TBST for 1h at RT, use 1/25,000 for low abundance samples.
7. Wash 4 X with TBST for 15min each.
8. Detect with KPL Inc.'s Chemiluminesence Detection Kit (KPL Inc. Cat. # 54-61-02 Tel: 800-638-3167), use a 30sec exposure to x-ray film and if necessary follow up with a 5min exposure to achieve a band in the lane containing the positive control. Developer (1min), water (1min), fixer (1min), water (2min). Hang-up to dry.

2. Immuno-cytochemistry

Reagents required

1) Primary antibody i.e. this product.
2) Secondary antibody e.g. anti-mouse rhodamine conjugate (Cat.# MG05).
3) Phosphate Buffered Saline (PBS, 50mM potassium phosphate pH 7.4, 50 mM NaCl).
4) PBS plus 3% BSA (Blocking solution)
5) Antifade mounting medium (e.g. Molecular Probes Inc. Cat# S-7461).
5) Glass mounting slide (25x75mm 1.0mm thick).
6) Coverslip sealing solution (usually a quick drying varnish).

Protocol

All procedures are at room temperature

1. Pour off culture media and wash cells once with PBS while coverslips are still in Petri dish. Use ISO-temp PBS so as not to disturb cells.
2. Fix cells either in 100% methanol at room temperature for 2min or formaldehyde/gluteraldehyde 3.0% / 0.5% in PBS for 1hr.
3. Wash once with PBS at room temperature for 30secs. Quench formaldehyde with 10mM Na Borohydride, and wash with PBS again (all steps are performed at room temperature from here on).
4. Place coverslips on parafilm
5. Block with 3% BSA in PBS for 60min.
6. Wash once with PBS.
7. Incubate in 1/200 dilution of this product, use 1ul antibody in 200ul PBS plus 3% BSA, incubate for 2h.
8. Wash three times in PBS plus 1% Triton X-100 (let stand for 5min each).
9. Incubate in anti-rabbit rhodamine (Cat.# MG05) at 1/500 dilution in PBS plus 3% BSA, again for 2h.

Continued on page 3 ...

2. Immuno-cytochemistry continued...

10. Wash three times in PBS (without Triton X-100), let stand for 5min each.
11. Mount in fixative with anti-fade and visualize under fluorescence microscope.

3. Immuno-precipitation (Not recommended)

Immunoprecipitation with talin is not recommended because the protein is large and generally insoluble, which does not allow sufficient amounts in the soluble form for Ip reactions.
