# Supplementary material for: Specific post-translational modifications of soluble tau protein distinguishes Alzheimer’s disease and primary tauopathies
Source: Nat Commun. 2023 Jun 22;14:3706. doi: 10.1038/s41467-023-39328-1 (PMC10287718; doi:10.1038/s41467-023-39328-1)
Supplement: Supplementary file 3 — Reporting Summary [file 41467_2023_39328_MOESM3_ESM.pdf]

nature portfolio

Corresponding author(s): Bernard Hanseeuw

Last updated by author(s): May 17, 2023

Reporting Summary

Nature Portfolio wishes to improve the reproducibility of the work that we publish. This form provides structure for consistency and transparency in reporting. For further information on Nature Portfolio policies, see our Editorial Policies and the Editorial Policy Checklist.

Statistics

For all statistical analyses, confirm that the following items are present in the figure legend, table legend, main text, or Methods section.

n/a	Confirmed	
☐	☒	The exact sample size (*n*) for each experimental group/condition, given as a discrete number and unit of measurement
☐	☒	A statement on whether measurements were taken from distinct samples or whether the same sample was measured repeatedly
☐	☒	The statistical test(s) used AND whether they are one- or two-sided<br>*Only common tests should be described solely by name; describe more complex techniques in the Methods section.*
☐	☒	A description of all covariates tested
☐	☒	A description of any assumptions or corrections, such as tests of normality and adjustment for multiple comparisons
☐	☒	A full description of the statistical parameters including central tendency (e.g. means) or other basic estimates (e.g. regression coefficient) AND variation (e.g. standard deviation) or associated estimates of uncertainty (e.g. confidence intervals)
☐	☒	For null hypothesis testing, the test statistic (e.g. *F*, *t*, *r*) with confidence intervals, effect sizes, degrees of freedom and *P* value noted<br>*Give P values as exact values whenever suitable.*
☒	☐	For Bayesian analysis, information on the choice of priors and Markov chain Monte Carlo settings
☒	☐	For hierarchical and complex designs, identification of the appropriate level for tests and full reporting of outcomes
☐	☒	Estimates of effect sizes (e.g. Cohen's *d*, Pearson's *r*), indicating how they were calculated

Our web collection on statistics for biologists contains articles on many of the points above.

Software and code

Policy information about availability of computer code

Data collection	Thermo Scientific™ SureQuant Tune (Version 3.5), Proteome Discoverer (version 2.5)
Data analysis	Skyline (version 21.1), R Studio (version 4.2.2)

For manuscripts utilizing custom algorithms or software that are central to the research but not yet described in published literature, software must be made available to editors and reviewers. We strongly encourage code deposition in a community repository (e.g. GitHub). See the Nature Portfolio guidelines for submitting code & software for further information.

Data

Policy information about availability of data

All manuscripts must include a data availability statement. This statement should provide the following information, where applicable:

- Accession codes, unique identifiers, or web links for publicly available datasets
- A description of any restrictions on data availability
- For clinical datasets or third party data, please ensure that the statement adheres to our policy

The untargeted MS proteomic data (PTMs) were made available for Reviewers via Proteome Exchange Pride repository (see the reviewer account details here below) and will be made freely accessible to the scientific community, should our manuscript be accepted for publication.

The human proteome database used for untargeted mass spectrometry analyses is available on Uniprot under the identifier UP000005640 [https://

www.uniprot.org/proteomes/UP000005640].

Targeted MS proteomic (SureQuant absolute quantification) raw quantification data will be made accessible upon request due to incompatibilities with the template of the approved and recommended data repositories. The corresponding quantification results are available as tables in the supplementary information and Source Data file file (sheet 1).

Project Name: Specific post-translational modifications of the soluble tau protein distinguish between Alzheimer's disease, 4R-, and 3R-tauopathies.
Project accession: PXD038901
Project DOI: 10.6019/PXD038901
Reviewer account details:
Username: reviewer_pxd038901@ebi.ac.uk
Password: 9yJQSCmD

Human research participants

Policy information about studies involving human research participants and Sex and Gender in Research.

Reporting on sex and gender

The sex of individual from whom brain tissue sample were collected was provided by the Netherlands Brain Bank (NBB):
Non-entendement control group (CTL) : 3 females + 2 males
Alzheimer's disease group (AD) : 8 females + 7 males
Corticobasal degeneration group (CBD) : 3 females + 2 males
Pick's disease group (PiD) : 5 males
Fronto-temporal dementia tauopathy group (FTLD): 3 females + 7 males
The female percentage were compared between the different groups using Fischer exact test ($P < 0.05$) and the results are reported in Table 1 of the manuscript.

Population characteristics

Common sample characteristics:

- Biospecimen type : solid tissue
- Anatomical site:brain
- Vital State : post mortem
- Collection mechanism: autopsy
- Type of stabilization: on ice
- Type of long-term preservation: freezing
- Storage temperature: −80 °C
- Shipping temperature: −78.5 °C (Dry ice)

Variable sample characteristics:

Sample # ; Pathology diagnosis ; Age at death (years) ; Post mortem delay (min) ; ApoE genotype ; brainweight (g) ; Braak stage ; amyloid (CERAD) ; Storage duration
1 ; AD ; 67 ; 455 ; 33 ; 817 ; 5 ; C ; 15 years
2 ; AD ; 83 ; 295 ; 43 ; 1100 ; 6 ; C ; 15 years
3 ; AD ; 82 ; 315 ; 44 ; 1182 ; 5 ; C ; 14 years
4 ; AD ; 82 ; 255 ; 43 ; 1205 ; 5 ; C ; 14 years
5 ; AD ; 81 ; 315 ; 43 ; 888 ; 6 ; C ; 14 years
6 ; AD ; 54 ; 395 ; 43 ; 867 ; 6 ; C ; 14 years
7 ; AD ; 74 ; 205 ; 44 ; 1200 ; 5 ; C ; 13 years
8 ; AD ; 65 ; 212 ; 33 ; 1139 ; 6 ; C ; 13 years
9 ; AD ; 57 ; 230 ; 43 ; 995 ; 6 ; C ; 13 years
10 ; AD ; 88 ; 405 ; - ; 1148 ; 5 ; C ; 12 years
11 ; AD ; 57 ; 345 ; 43 ; 960 ; 6 ; C ; 10 years
12 ; AD ; 70 ; 240 ; - ; 870 ; 6 ; C ; 5 years
13 ; AD ; 68 ; 320 ; 43 ; 1100 ; 6 ; C ; 14 years
14 ; AD ; 58 ; 315 ; 33 ; 985 ; 6 ; C ; 13 years
15 ; AD ; 77 ; 365 ; 33 ; 999 ; 5 ; C ; 15 years
16 ; CBD ; 73 ; 370 ; 33 ; 1293 ; - ; - ; 11 years
17 ; CBD ; 72 ; 320 ; 33 ; 1130 ; - ; - ; 10 years
18 ; CBD ; 65 ; 400 ; - ; 957 ; - ; - ; 8 years
19 ; CBD ; 58 ; 420 ; - ; 980 ; 0 ; A ; 7 years
44 ; CBD ; 70 ; 3000 ; - ; - ; - ; - ; 2 years
20 ; PiD ; 57 ; 400 ; 33 ; 897 ; - ; - ; 16 years
21 ; PiD ; 64 ; 345 ; - ; 1049 ; - ; - ; 13 years
22 ; PiD ; 60 ; 375 ; 33 ; 1445 ; - ; - ; 11 years
23 ; PiD ; 60 ; 315 ; - ; 872 ; - ; - ; 8 years
24 ; PiD ; 67 ; 480 ; - ; 915 ; 0 ; O ; 5 years

25	FTLD	54	340	33	791	-	-	18 years
26	FTLD	67	250	43	757	-	-	18 years
27	FTLD	64	310	43	952	-	-	18 years
29	FTLD	54	450	43	1027	-	-	17 years
30	FTLD	75	406	33	875	-	-	16 years
31	FTLD	49	295	43	1068	-	-	14 years
32	FTLD	61	360	-	740	-	-	13 years
35	FTLD	65	240	-	1000	-	-	13 years
36	FTLD	57	455	-	1111	-	O	6 years
37	FTLD	55	265	-	1100	0	O	6 years
39	CTL	87	300	-	1010	2	O	13 years
40	CTL	76	285	-	1140	2	-	6 years
41	CTL	72	260	-	1385	2	-	4 years
42	CTL	81	475	33	1194	2	O	14 years
43	CTL	78	430	33	1120	1	A	7 years

Recruitment: Netherlands Brain Bank (NBB)

Ethics oversight: Ethical and legal declaration of the Netherlands Brain Bank (see joint document)

Note that full information on the approval of the study protocol must also be provided in the manuscript.

Field-specific reporting

Please select the one below that is the best fit for your research. If you are not sure, read the appropriate sections before making your selection.

- [x] Life sciences
- [ ] Behavioural & social sciences
- [ ] Ecological, evolutionary & environmental sciences

For a reference copy of the document with all sections, see nature.com/documents/nr-reporting-summary-flat.pdf

Life sciences study design

All studies must disclose on these points even when the disclosure is negative.

Sample size

No statistical method was used to predetermine sample size. For primary tauopathies, sample sizes were based on the availability of post-mortem brain tissue (n= 5 PiD, n=5 CBD, n=10 FTLD-tau). We then selected 5 control cases and 15 AD cases to have a minimum of 5 individuals per group and provide sufficient variability in the ratio of 4R-/3R-tau isoforms. A minimal sample size of n=5 per group was deemed sufficient as it allows observing significant differences between groups for tau isoforms in the aggregates. A total sample of n=40 was deemed sufficient as it allows disclosing correlations between PTMs and the ratio of 4R-/3R-tau isoforms when those variables share at least 10% of variance (R2 > 0.10, P < 0.05).

Data exclusions

Data from sample 34 were excluded from all analyses for technical reasons (insufficient biological material).

Data from samples 28, 33 and 38 (FTLD group) were excluded from downstream analysis due to the impossibility of classification as either "3R" or "4R" tauopathy required for this study.

Replication

MS data were aquired once for each subject per group and each type of aquisition. Each Groups comprised n subjects (biologically independent samples/biological replicates): controls group, n=5; AD group, n=15; CBD group, n=5; FTLD group n=10; PiD group, n=5.

No technical replicate was perform on this work.

Randomization

Subjects were not allocated to randomized groups. This is not a clinical trial.

Blinding

Irrelevant because no randomization was conducted.

Reporting for specific materials, systems and methods

We require information from authors about some types of materials, experimental systems and methods used in many studies. Here, indicate whether each material, system or method listed is relevant to your study. If you are not sure if a list item applies to your research, read the appropriate section before selecting a response.

Materials & experimental systems

n/a	Involved in the study
☐	☒ Antibodies
☒	☐ Eukaryotic cell lines
☒	☐ Palaeontology and archaeology
☒	☐ Animals and other organisms
☒	☐ Clinical data
☒	☐ Dual use research of concern

Methods

n/a	Involved in the study
☒	☐ ChIP-seq
☒	☐ Flow cytometry
☒	☐ MRI-based neuroimaging

Antibodies

Antibodies used

Antibody ; Antibody ID ; Supplier name ; Catalog number ; Clone name ; Lot number

(1) Mouse mAb HJ8.7 ; AB_2721234 [http://antibodyregistry.org/AB_2721234] ; D. Holtzman at Washington University School of Medicine, Missouri, USA ; HJ8.7 AB_2721234 ; Not mentioned

(2) Rabbit mAb Tau (D1M9X) XP® ; Tau (D1M9X) XP [https://media.cellsignal.com/coa/46687/1/46687-lot-1-coa.pdf] ; Cell Signaling Technology (CST) ; # 46687 ; D1M9X ; Lot 01

(3) Mouse mAb Anti-Vinculin ; Mouse mAb VIN-11-5 ; Cytoskeleton Inc. ; # AVN01 ; VIN-11-5 ; Lot 025 (See supplementary Information file)

Validation

(1) Mouse mAb HJ8.7 ; PMID:24075978, PMID:29566794

(2) Rabbit mAb Tau (D1M9X) XP® ; PMID: 35993331, PMID: 34593029

(3) Mouse mAb Anti-Vinculin ; Discontinued, manufacturer data sheet available in Supplementary Information (The antibody can be provided by authors).
